# Cytomegalovirus and HIV Co-Infections During Pregnancy and Early Childhood

**DOI:** 10.1007/s11904-026-00788-x

**Published:** 2026-06-23

**Authors:** Swetha Pinninti, Nitin Arora, Suresh Boppana

**Affiliations:** 1 Department of Pediatrics, Heersink School of Medicine, University of Alabama at Birmingham, Birmingham, AL USA; 2 Department of Microbiology, Heersink School of Medicine, University of Alabama at Birmingham, Birmingham, AL USA

**Keywords:** HIV, Cytomegalovirus, Congenital CMV, Sensorineural hearing loss, Development, HIV-exposed, HIV-infected, Co-infections in pregnant women

## Abstract

**Purpose of Review:**

This review summarizes the epidemiology of Cytomegalovirus infections in children exposed to or infected with HIV and the impact of CMV infection on overall health outcomes in this population.

**Recent Findings:**

CMV infections and cCMV remain a significant burden in HIV exposed and HIV infected children requiring close long-term follow-up.

**Summary:**

While CMV disease occurs rarely in HIV-infected individuals in the ART-era, the complex interplay between CMV and HIV could have a major impact on childhood health outcomes in HIV-infected and exposed children. Congenital CMV infection (cCMV) is well recognized as the most common congenital viral infection and the leading cause of sensorineural hearing loss (SNHL). CMV-HIV co-infections have been shown to increase the incidence of cCMV, but effect on long-term outcomes remains largely undetermined at this time.

## Introduction

Human cytomegalovirus (CMV) is a large double-stranded DNA virus belonging to the beta herpesvirus family. CMV infections are ubiquitous with seroprevalence rates ranging from 50 to > 90% [1–5] (Table 1). Like other herpesviruses, CMV infections are life-long, persistent infections and can be acquired at any age. However, acquisition via breast milk is the most common mode of transmission, especially in low and middle-income countries (LMIC) [3].

**Table 1:** Cytomegalovirus seroprevalence data*

Region	CMV seroprevalence in women of reproductive age (%)
Africa	60–100 Women living with HIV: 77.3–100
Europe	45.6–95.7
Japan	60.2
Latin America	58.3–94.5
US and Canada	24.6–81.0

^*^[4–6]

**Fig. 1:**
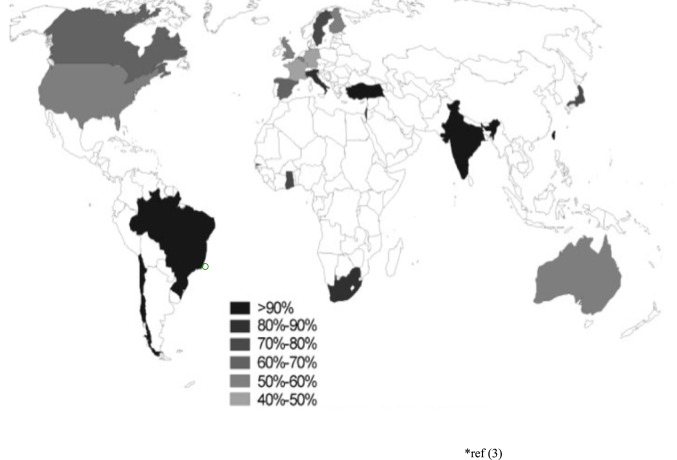
Worldwide CMV seroprevalence rate in women of childbearing age*

Worldwide, Human Immunodeficiency Virus (HIV) remains one of the most significant public health challenges. In 2023, approximately 1.2 million pregnant women were infected with HIV globally, of whom, an estimated 84% received antiretroviral drugs to prevent mother-to-child transmission. As of the end of 2024, there were 1.4 million children living with HIV globally, down from 2.7 million in 2010. The success of mother-to-child transmission programs over the past several decades has led to an increasing number of infants who are exposed to HIV but remain uninfected (Fig. 1).

CMV infections cause end-organ disease with significant morbidity and mortality in immunocompromised individuals such as allograft recipients and those with HIV/AIDS with CD4 < 50. Although CMV disease in HIV-infected individuals has become rare in the antiretroviral therapy (ART) era, the complex interaction between CMV and HIV infections likely plays an important role in overall health outcomes, especially in children. CMV is also a common cause of congenital infection (mother-to-fetus transmission) and congenital CMV infection (cCMV) is a leading cause of non-hereditary sensorineural hearing loss (SNHL) and adverse neurodevelopmental outcomes in children worldwide [1, 2]. Unlike other congenital infections like rubella, paradoxically, higher prevalence of cCMV has been documented in populations with high CMV seroprevalence [7] (Table 2). In addition, intrauterine transmission of CMV can occur following both primary (initial infection during pregnancy) and non-primary maternal CMV infections. Higher rates of cCMV have been reported in infants born to mothers with HIV and this increase in CMV transmission rates persists despite mothers receiving ART. This review summarizes the epidemiology and natural history of cCMV in infants born to mothers with HIV infection and the impact of CMV infection on overall health outcomes in HIV-exposed (both infected and exposed) infants.

**Table 2:** Congenital cytomegalovirus incidence rates*

Region	cCMV Incidence - % (publications since 2016)
High-Income Countries (HICs)	
Japan	0.31
France	0.38
Norway	0.22
Finland	0.2
Japan	0.48
US	0.45
Low- to Middle-Income Countries	
Iran	0.49
China	0.69
Mozambique	5.98
Indonesia	5.84
India	0.4
South Africa	2.46
Kenya	3.62
Bosnia and Herzogovina	0.62
Iran	0.34
Brazil	0.57
Colombia	0.84
Gambia	5.4
Ivory Coast	1.4

^*^[7]

### CMV Seroprevalence and CMV-HIV Co-Infections In Pregnant Women

Age-related prevalence of CMV infection in the population varies widely depending on socio-economic status, overcrowding and social customs. In resource-limited settings, most individuals acquire CMV early during childhood [8]. In a systematic review and meta-analysis, global CMV seroprevalence was estimated to be 83% (95% CI: 78–88) in the general population, with highest rates in the WHO Eastern Mediterranean region compared to the European region (90% vs 66%). Among women of childbearing age and donors of blood and organs, CMV seroprevalence was estimated to be 86% (95% CI: 83–89) with a strong association with socio-economic status [9]. Among U.S. women of childbearing age, the prevalence of CMV seropositivity is estimated to be 30 to 70%, with strong correlations to socioeconomic status and household income [6, 10]. CMV seroprevalence is higher than in the general population among pregnant women with HIV, with HIV-CMV co-infections estimated in approximately 90% of pregnant women [11, 12].

### Maternal HIV and Congenital Cytomegalovirus Infection (cCMV)

CMV shedding in bodily fluids (saliva, urine, breast milk, cervical secretions) in CMV seropositive individuals and shedding from young children is considered a leading source for CMV exposure for primary and non-primary CMV infections for pregnant women [13, 14]. Additionally, STD clinic attendance and co-infections have been shown to be associated with increased incidence of CMV shedding [15–17].

Similarly, among CMV-seropositive women, higher rates of cervical CMV shedding are observed in women with HIV-CMV co-infections than in women without HIV (52% vs 14–35%) [18]. Additionally, higher cCMV incidence has been documented in babies born to HIV-infected pregnant women and with in-utero HIV infection, compared to HIV-exposed, uninfected infants (18% vs 4.9%), with in-utero HIV transmission [19]. Higher rates of cCMV have also been shown in HIV-exposed, uninfected infants (1.5% - 5.2%) compared to unexposed infants, suggesting HIV-CMV co-infections facilitate maternal–fetal CMV transmission, similar to other STI’s [7, 11, 19–25]. In addition, infants with cCMV infection exposed to HIV have a 20-fold higher risk for HIV co-transmission compared to infants without cCMV infection [23].

In the ART era, prevalence of cCMV infection in infants born to mothers with HIV has ranged from 2.2% to 5.2% [11, 20–23] (Table 3). Data from a high–HIV prevalence areas in the ART era suggested that *in utero* HIV transmission is significantly higher in neonates with cCMV than without cCMV infection (OR: 20.1, 95% CI, 6.09–66.46) [23]. Higher proportion of infants with HIV-cCMV co-infection experience symptomatic cCMV disease compared to HIV exposed but uninfected infants (23.1% vs 6.7%), despite ART [20]. The clinical presentations in infants with symptomatic cCMV with or without HIV include small for gestational age, petechiae, jaundice, hepatosplenomegaly, chorioretinitis, microcephaly, intracranial calcifications, and sensorineural hearing loss (SNHL). However, it remains unclear whether infants with vertically transmitted, asymptomatic cCMV co-infected with HIV experience high rates of long-term CMV-related sequelae than those without HIV coinfections.

**Table 3:** Congenital cytomegalovirus incidence rates in HIV-CMV co-infections in ART era

Region	cCMV incidence (%)	Reference
France	2.3 HIV infected neonates – 10.3 HIV uninfected neonates – 2.2	Guibert et al. [20]
US	3	Duryea et al. [24]
South Africa	2.9	Manicklal et al. [21]
US	2.2	Gantt et al. [22]
South Africa	5.2	Pathirana et al. [23]
US	2.5	Smith et al. [11]

To summarize, the existing data demonstrates higher cCMV prevalence among HIV-exposed infants compared with those unexposed, higher rates of CMV and HIV co-transmission, and increased frequency of symptomatic cCMV infection among infants co-infected with HIV.

### Maternal–Fetal Interface in HIV and CMV Infections

A Canadian cohort of women living with HIV revealed that cord marginality (distance from the edge) was significantly lower in the HIV positive group, with 35% of placenta having an abnormal (marginal or velamentous) cord insertion compared to 12.5% in the HIV negative group [26]. A Kenyan study compared placentas WLHIV who had preterm deliveries with placentas of HIV-negative women. In preterm placentas, HIV was significantly associated with thrombosis, infarction, anomalies in cord insertion, gross evidence of membrane infection, and reduced placental thickness. The features suggested that HIV infection may cause morphological changes in placenta which could result in preterm delivery [27].

Early studies reported an increased proportion of acute chorioamnionitis and funisitis without villitis in women with HIV in the pre-ART era, likely from HIV related inflammation [28]. A study in South Africa found that maternal vascular malperfusion was more common in placentas of women with HIV than HIV-negative women [29]. In addition, women with HIV receiving combination ART had more placental infarcts than women on Zidovudine [29]. Another South African study found women with HIV had lower placental weight and more marginal infarcts than HIV-negative women, and that villitis of unknown etiology was the most notable finding in women living with HIV [30]. Another study found that chronic placental inflammation was not associated with HIV, and VUE was twice as common in HIV-negative women than women living with HIV. In addition, an increased number of CD3, CD20, and CD68 positive cells in villous staining in HIV-negative women than women living with HIV, suggesting dysregulated immune inflammatory responses in women living with HIV [31]. While there is no clear consensus on placental lesions associated with HIV, acute chorioamnionitis, maternal vascular malperfusion, and decreased placental weight appear to be common features.

Gross examination of a CMV infected placenta may reveal edema with membranous opacities [32]. Histological assessment identifies characteristic cytomegalic cells with enlarged nuclei and CMV intracellular inclusions [33]. Typical histological findings of CMV infection include chronic villitis, viral inclusions, hemosiderin deposition and plasma cell infiltration [34]. The latter may be seen with a variety of placental inflammatory conditions and infections but is especially common in placental CMV infection [33–36].

### Post-Natal CMV Acquisition in HIV Exposed and Infected Infants

CMV acquisition from breast milk is the most common route of post-natal CMV acquisition. In a cohort of CMV seropositive pregnant women followed throughout pregnancy and the post-partum period, CMV shedding from mucosal compartments was documented in 35% of the cohort and shed CMV five times more frequently in breast milk than any other specimen [37]. In regions with high rates of maternal seropositivity and breastfeeding practices, more than half of infants acquire CMV postnatally during the first year after birth [22, 38, 39]. Additionally, group childcare centers facilitate CMV spread especially among toddlers, and can lead to higher prevalence of infection in children who attend childcare centers and, in their caregivers [14, 40, 41].

However, symptomatic CMV disease and cCMV-associated long-term sequelae are uncommon in children with post-natal acquisition except in extremely premature infants, who experience CMV disease including persistent thrombocytopenia, pneumonitis, hepatitis and/colitis, leading to significant morbidity [42–45]. The risk of acquiring CMV infection during early childhood appears to be greater for children with HIV than for children without HIV (39.9% vs. 15.3%) and is particularly high in the first 12 months of life [39, 46, 47].

In the pre-ART era, among HIV-infected children, HIV disease has been shown to progresses more rapidly in those co-infected with CMV than in those without CMV infection [48–51]. The relative risk of HIV disease progression in children co-infected with CMV compared to children without CMV is 2.6 (95% CI, 1.1–6.0) [48]. However, more recent data, albeit limited, indicate that infants with HIV-CMV co-infection on ART may not experience accelerated HIV disease progression [52, 53].

### Impact of CMV-HIV Co-Infection on Clinical Outcomes

In the ART-era, the frequency of CMV disease has been shown to decline among children with HIV. However, CMV co-infection has been associated with an adverse impact on the general health of children exposed to or with HIV. In a cohort of Kenyan children who were initiated on ART prior to 12 months of age, high pre-ART HIV viral load and CMV viremia in infancy were associated with lower executive function and cognitive ability scores [54]. Among older children and adolescents with perinatally acquired HIV, the frequency of CMV DNAemia was significantly higher in HIV infected ART-naïve and ART-treated children, compared to HIV-uninfected controls and associated with reduced lung function and stunting, respectively, suggesting a role for CMV in the pathogenesis HIV comorbidities [55]. Additionally, CMV co-infection in HIV exposed or infected infants has been shown to be associated with immunological changes (alteration in CD8 + T-cell compartment), albeit of unknown significance [56, 57].

### CMV Disease in Children with HIV

In the pre-ART era, approximately 25% of all AIDS-defining illnesses were attributable to CMV retinitis, the most frequent end-organ disease among children with HIV. However, in the ART era, CMV retinitis rate in children with HIV has significantly decreased to 0–0.4% [58, 59]. In the Perinatal AIDS Collaborative Transmission Study, the incidence of non-ocular CMV disease before and after January 1997 (pre-ART and ART eras) declined from 1.4 per 100 child-years to 0.1 per 100 child-years, with CMV retinitis rates declining from 0.7 to 0.0 per 100 child-years; with a decreasing trend in opportunistic infections that continued in the next decade after introduction of ART [60, 61].

End-organ CMV disease, involving lung, liver, gastrointestinal (GI) tract, pancreas, kidney, and CNS have been demonstrated in children with HIV, but is rare in the current ART era. Gastrointestinal (GI) manifestations among children with HIV classically included CMV colitis (the most common GI manifestation), oral and esophageal ulcers, hepatitis, ascending cholangiopathy, or gastritis. Odynophagia was a frequent presentation of CMV esophagitis, while abdominal pain and hematochezia frequently occurred in children with CMV colitis. Visualization of intestinal lumen in affected children frequently revealed nonspecific findings including diffuse erythema, submucosal hemorrhage, and diffuse mucosal ulcerations.

The role of CMV in pulmonary disease among children with HIV is difficult to assess because CMV often is found along with other organisms (e.g., *Pneumocystis jirovecii*). Determination of active disease requires histological evidence of CMV disease. CMV pneumonia is an interstitial process with gradual onset of shortness of breath and dry, nonproductive cough; auscultatory findings may be minimal.

CNS manifestations of CMV disease include subacute encephalopathy, myelitis, and polyradiculopathy (primarily observed in adults but rarely reported in children). Cerebrospinal fluid (CSF) findings are nonspecific and may include leukocytosis with polymorphonuclear predominance, elevated protein, and low glucose. Occasionally, CMV can cause a rapidly progressive, often fatal CNS disease with cranial nerve deficits, nystagmus, and increasing ventricular size.

## Conclusion

The landscape of HIV infections has drastically changed since the introduction of anti-retroviral therapy with improved long-term outcomes and decreased incidence of opportunistic infections. However, CMV seroprevalence rates remain high in pregnant women with HIV, leading to higher prevalence of cCMV among HIV-exposed infants, higher rates of CMV and HIV co-transmission, and increased frequency of symptomatic cCMV infection among infants co-infected with HIV. The long-term outcomes of this co-infection in affected children remains largely unknown and needs further evaluation to develop effective interventions.

## Key References

- Ssentongo P, Hehnly C, Birungi P, Roach MA, Spady J, Fronterre C, et al. Congenital Cytomegalovirus Infection Burden and Epidemiologic Risk Factors in Countries With Universal Screening: A Systematic Review and Meta-analysis. JAMA Netw Open. 2021;4(8):e2120736.
  - ○ A systematic review and meta-analysis that details cCMV prevalence identified through universal screening from across the globe and highlights the burden of cCMV in low and middle income countries.
- Nesheim SR, Balaji A, Hu X, Lampe M, Dominguez KL. Opportunistic Illnesses in Children With HIV Infection in the United States, 1997-2016. Pediatr Infect Dis J. 2021;40(7):645-8
  - ○ This study highlights the drastic decrease in opportunistic infections in HIV infected children in the US after introduction of anti-retroviral therapy.
- Fowler K, Mucha J, Neumann M, Lewandowski W, Kaczanowska M, Grys M, et al. A systematic literature review of the global seroprevalence of cytomegalovirus: possible implications for treatment, screening, and vaccine development. BMC Public Health. 2022;22(1):1659.
  - ○ This systematic review provides an overview of the global burden of CMV.
- Fougère Y, Brophy J, Hawkes MT, Lee T, Samson L, Gantt S, et al. Clinical and Immunologic Impact of CMV Coinfection Among Children Living With HIV in Canada. Pediatr Infect Dis J. 2025;44(8):764-71.
  - ○ This study highlights the distinct immunological changes in children co-infected with HIV and CMV

## Data Availability

No datasets were generated or analysed during the current study.
